# Short-term trends in the prevalence, awareness, treatment, and control of arterial hypertension in Peru

**DOI:** 10.1038/s41371-020-0361-1

**Published:** 2020-06-09

**Authors:** David Villarreal-Zegarra, Rodrigo M. Carrillo-Larco, Antonio Bernabe-Ortiz

**Affiliations:** 1 CRONICAS Center of Excellence in Chronic Diseases, Universidad Peruana Cayetano Heredia, Lima, Peru; 2 Department of Epidemiology and Biostatistics, School of Public Health, Imperial College London, London, UK; 3 Instituto de Investigación, Universidad Católica de Trujillo, Chimbote, Peru; 4 Universidad Científica del Sur, Lima, Peru

**Keywords:** Risk factors, Hypertension

## Abstract

The prevalence of hypertension has been declining in low- and middle-income countries (LMIC), particularly in Latin America and the Caribbean. However, we have not identified studies that evaluate trends for awareness, treatment, and control of hypertension in LMIC. We aimed to describe the trends in the prevalence, awareness, treatment, and control of hypertension in Peru. A cross-sectional analysis was conducted using secondary data (4 years) of the Demographic and Health Survey of Peru (ENDES, Spanish acronym), which is conducted annually and is representative at the country level. The age-standardized prevalence was estimated using the World Health Organization population as the reference population. The trend over time was evaluated with the score test for trend of odds. A total of 109,401 participants were included. In Peru, from 2015 to 2018, the age-standardized prevalence of hypertension increased (*p* < 0.001), while the proportion of people with disease awareness (*p* < 0.001) and controlled hypertension decreased (*p* = 0.01). During that same period, the proportion of people with treatment for hypertension did not vary over time (*p* = 0.13). In 2018, the age-standardized prevalence of hypertension was 20.6%, and the proportion of people with disease awareness, treatment, and control of arterial hypertension was 43.5%, 20.6%, 5.3%, respectively. People with low socioeconomic status and people living in rural areas have the lowest proportion of awareness, treatment, and control of hypertension.

## Background

Hypertension is one of the most important risk factors for different cardiovascular diseases [1]. Providing greater access to treatment and developing policies to treat hypertension has proven to be a cost-effective intervention that would reduce the number of deaths due to cardiovascular diseases and a large number of cardiovascular problems [2]. In particular, ~80% of all deaths in low- and middle-income countries (LMIC) are due to cardiovascular disease [3].

The prevalence of hypertension is around 22% in LMIC; however, only 43.6% of the cases are aware of having this condition, 36.9% receive appropriate treatment, yet only 9.9% are controlled [4]. The prevalence of high blood pressure has been declining in LMIC, particularly in Latin America and the Caribbean [5]. However, we have not identified studies that evaluate trends for awareness, treatment, and control of hypertension in LMIC.

Identifying current trends and distributions of awareness, treatment, and control of hypertension will allow a better allocation of resources, including relevant policies and interventions in LMIC. A study identified that in 12 high-income countries, the awareness, treatment, and control of hypertension improved substantially, and the prevalence of hypertension reduced [6]. One potential reason for this is that high-income countries have higher health budgets and have implemented programs from chronic disease detection and control [6, 7].

The use of trend analysis methodology to measure changes in prevalence, awareness, treatment, and control of hypertension at the national level was originally done using the National Health and Nutrition Examination Survey [8]. Currently, other studies have used the same methodology to test this aim in different countries as these three indicators (i.e., awareness, treatment, and control) vary according to the characteristics of each country. National studies in China and Korea suggest that these measures are trending upward in all areas [9, 10]. In comparison, a national study in Chile suggests that the percentage of people with disease awareness remains stable over time, but the percentage of people with treatment and control have increased [11]. In Mexico, awareness, treatment, and control have all stabilized [12]. Further work to identify trends over time will allow a more complete assessment of whether national health systems are improving access to health. Therefore, we aimed to describe the short-term trends in the prevalence, awareness, treatment, and control of hypertension in Peru.

## Methods

### Study design

Data from the National Demographic and Health Survey of Peru (ENDES—Spanish acronym), which collects information on several variables including poverty, fertility, violence, and health, was utilized in this study. Our study, therefore, decided to use data collected by ENDES over these 4 years (between the years 2015 and 2018).

### Study population and selection criteria

The sampling used a bietapic approach. In rural areas, the primary sampling units consisted of clusters of 500–2000 individuals, and the secondary sampling units were households within each of these clusters. In urban areas, the sampling units consisted of blocks or groups of blocks with more than 2000 individuals and an average of 140 households, and secondary sampling units consisted of households as in rural settings [13]. Details on the sampling process can be found in the technical documents of the ENDES [14]. This study only included participants with complete blood pressure measurements (systolic and diastolic) and information on sociodemographic variables of interest (sex, age, area, economic level, year of evaluation, and region of origin).

### Procedures

#### Data collection process

Blood pressure levels were collected using a digital tensiometer (OMRON, model HEM-713). Two types of cuffs were used, depending on the arm circumference of the participants: standard arm (220–320 mm) and thicker arm (330–430 mm). The blood pressure measurements were evaluated twice with the participant sitting and their right arm resting on a flat surface at the heart level. The first measurement was taken after a rest period of 5 min, and the second measurement was taken 2 min after the first measurement [15]. In addition, ENDES collected self-reported information about whether the participant had a previous diagnosis of hypertension, received antihypertensive medication, and other sociodemographic data.

#### Assessment of consistency and plausibility of measurements

To ensure that only plausible blood pressure measurements were included in the analysis, a cut-off point was used as in pooled studies [5]: systolic blood pressure (SBP) measurements should be ≤270 mmHg and ≥70 mmHg; and the diastolic blood pressure (DBP) measurements should be ≤150 mmHg and ≥50 mmHg [5]. The no-plausible measures were dropped from analyses.

We defined three criteria to ascertain the consistency between the two blood pressure measurements. First, if the difference between the two measures of SBP (or DBP) was ≤5 mmHg, both measures were averaged. Second, if the difference between two of the SBP (or DBP) measurements was ≥6 mmHg, only the second blood pressure measurement was used, provided that the first measurement was greater than the second. This criterion arises because the evidence indicates that the first measurement is often greater than the second because of the white coat effect [16]. Third, if the difference between the two measurements of SBP (or BPD) was ≥6 mmHg, and the second measure was greater than the first, this observation was excluded from the analysis. We use this criterion because in epidemiological studies the first measurement tends to be greater than the second measurement of blood pressure [16, 17].

### Variables

#### Hypertension

Hypertension was defined as those having SBP ≥ 140 mmHg or DBP ≥ 90 mmHg, or having a previous self-reported diagnosis of hypertension.

#### Hypertensive patients with disease awareness

Participants aware of hypertension were those who reported having received a previous diagnosis of hypertension.

#### Hypertensive patients with treatment

Participants aware of their hypertension status, and that self-reported taking appropriate antihypertensive medication.

#### Controlled hypertension

Participants aware of their hypertension status, that self-reported taking appropriate antihypertensive medication, and has normal blood pressure values (SBP < 140 mmHg and DBP < 90 mmHg).

#### Other variables

In addition, a set of sociodemographic variables were also considered. The socioeconomic status (SES) was defined in quintiles (very low, low, middle, high, and very high) based on a wealth index available in the ENDES [18]. This index was calculated based on assets and services that the participant reports having in their household. The calculation of this index can be consulted in the DHS Report [18]. Age, as a continuous variable, was split into groups of 10 years (15–24, 25–34, 35–44, 45–54, 55–64, 65–74, and 75 to more). The sex (male vs. female), study area (urban/rural), and year of ENDES evaluation were also included.

### Statistical methods

All statistical analyses were performed using Stata 13 (StataCorp, College Station, TX, USA) and the graphics were elaborated using the ggplot libraries in R (version 3. 5. 1) and QGIS v2.18. The number and percentage of participants dropped from analyses were reported. The analysis was performed for each year of measurement. The age-standardized prevalence was calculated taking as a reference from the population of the World Health Organization [19]. All the analyses took into account the weighting by complex sampling, and the “svy subpop” command was used for subgroup analyses [20]. The 95% confidence interval (95% CI) was calculated for the regional and national measures. The trend over time was evaluated with the score test for trend using the year 2015 as the reference category.

A sub-analysis was performed considering the cut-points recommended by the guidelines of the American College of Cardiology/American Heart Association (ACC/AHA) in 2017, where hypertension was defined as those with SBP ≥ 130 mmHg or DBP ≥ 80 mmHg [21].

## Results

### Participants

**Fig. 1:**
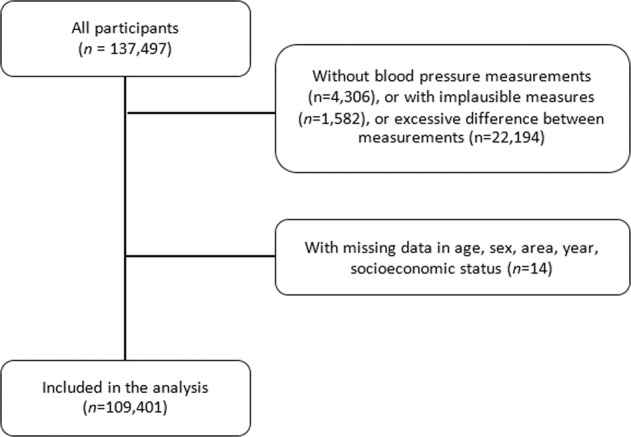
Flowchart of the participants included in our study. n number of participants.

From 2015 to 2018 a total of 137,497 individuals were evaluated in the ENDES. After applying the inclusion criteria of the study, 109,401 participants were included (see Fig. 1) and 28,096 participants were dropped (17.4% of the total, see Supplementary 1). The ages of the participants ranged between 15 and 98 years and the average age was 40.5 (SD: 16.7). Most of the participants were women (51.3%), were between 15 and 34 years old (43.4%), and lived in urban areas (72.6%). The characteristics of the participants included per year of evaluation can be seen in Table 1.

**Table 1:** Descriptive characteristics of the participants included in the study.

	2015		2016		2017		2018	
	*n*	%	*n*	%	*n*	%	*n*	%
Sex								
Male	12,217	48.9%	11,680	49.1%	11,811	48.4%	12,004	48.3%
Female	15,195	51.1%	15,000	50.9%	15,331	51.6%	16,163	51.7%
Age								
15–24	5,616	24.1%	5,295	23.4%	5,222	22.3%	5,228	21.4%
25–34	7,894	20.5%	7,394	20.5%	7,609	20.7%	7,847	20.7%
35–44	5,757	18.5%	5,630	18.6%	5,611	19.5%	6,196	19.4%
45–54	3,190	15.1%	3,242	15.2%	3,288	15.3%	3,355	15.3%
55–64	2,362	10.1%	2,408	10.4%	2,467	10.3%	2,646	10.9%
65–74	1,576	6.7%	1,588	6.7%	1,732	6.9%	1,793	7.1%
75+	1,017	5.1%	1,123	5.1%	1,213	5.0%	1,102	5.2%
Area								
Urban	17,998	65.5%	17,545	64.8%	17,925	79.5%	18,476	80.4%
Rural	9,414	34.6%	9,135	35.2%	9,217	20.5%	9,691	19.6%
SES								
Very low	7,668	26.2%	7,369	26.3%	8,139	18.3%	8,955	18.7%
Low	6,883	21.3%	7,005	22.2%	6,988	20.7%	6,979	20.8%
Middle	5,304	18.3%	5,243	18.5%	5,238	21.0%	5,232	20.7%
High	4,180	17.9%	4,188	17.4%	3,974	20.3%	3,991	20.3%
Very high	3,377	16.3%	2,875	15.7%	2,803	19.8%	3,010	19.5%
Hypertension^a^								
Yes	4,563	18.7%	4,445	18.5%	4,590	19.4%	5,168	20.6%
No	22,849	81.3%	22,235	81.5%	22,552	80.6%	22,999	79.4%

The weighted proportion by complex sampling.

*SES* socioeconomic status.

^a^Age-standarized prevalence.

### Prevalence of hypertension

**Fig. 2:**
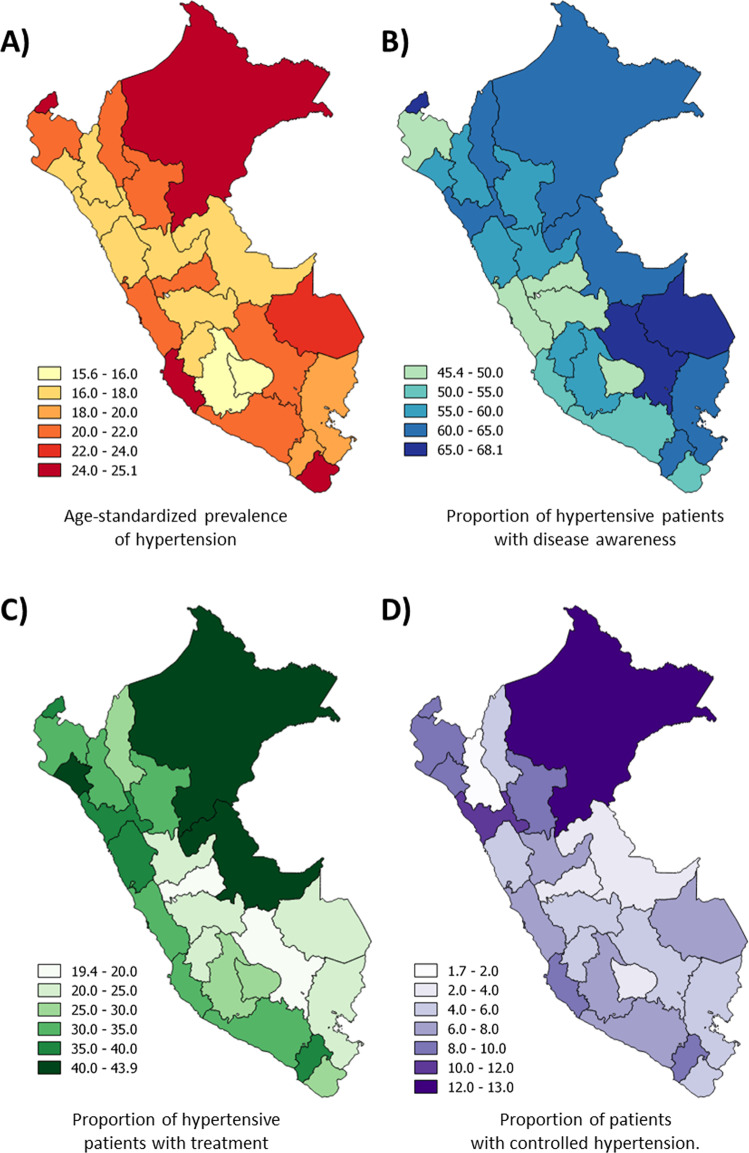
Age-standardized prevalence of hypertension, and the proportion of hypertensive patients with disease awareness and treatment and control by region in 2018. **a** Age-standardized prevalence of hypertension in Peru in 2018. **b** The proportion of hypertensive patients with disease awareness in Peru in 2018. **c** The proportion of hypertensive patients with treatment in Peru in 2018. **d** The proportion of patients with controlled hypertension in Peru in 2018.

The age-standardized prevalence of hypertension increased from 18.7% to 20.6% between 2015 and 2018 (score test for trend: *p* value < 0.001). Adults older than 75 years were the age group with the highest age-standardized prevalence (65.5% in 2018). Men (18.6% in 2015 to 22.4% in 2018) had a higher age-standardized prevalence compared to women (18.8% in 2015 and 2018). The age-standardized prevalence increased most in individuals with low SES (18.7% in 2015 to 21.9%) and meager SES (15.3% in 2015 to 16.0% in 2018). Age-standardized prevalence of hypertension (in 2018) was higher in the jungle and southern coastal provinces. The distribution by region can be seen in Fig. 2a.

### Hypertensive patients with disease awareness

Among participants with hypertension, the proportion of people aware of their condition reduced from 53.4% in 2015 to 43.5% in 2018 (score test for trend: *p* value < 0.001). A higher proportion of hypertensive individuals aware of their condition were women, and the proportion increased with age (see Table 2). In five of the 25 regions of Peru, the proportion of people who are aware of their disease was less than 50% (see Fig. 2b).

**Table 2:** Proportion of Peruvian hypertensive patients with disease awareness, treatment, and controlled hypertension by sociodemographic characteristics (2015–2018).

	2015	2016	2017	2018
Hypertensive patients with disease awareness				
Sex				
Male	45.3%	41.9%	37.9%	38.6%
Female	73.7%	69.2%	67.6%	70.5%
Age				
15–24	43.1%	27.4%	37.4%	33.6%
25–34	47.3%	35.7%	42.5%	33.4%
35–44	55.4%	50.7%	45.1%	40.8%
45–54	60.6%	54.6%	47.2%	50.1%
55–64	62.4%	57.7%	54.0%	58.1%
65–74	63.3%	62.1%	60.0%	63.5%
75+	66.2%	64.1%	62.6%	69.3%
Area				
Urban	62.6%	56.0%	53.1%	53.9%
Rural	54.1%	53.1%	49.8%	54.0%
SES				
Very low	50.4%	48.0%	46.5%	51.2%
Low	58.0%	56.6%	52.9%	52.2%
Middle	58.0%	58.8%	57.7%	52.5%
High	65.9%	53.8%	50.8%	55.7%
Very high	68.0%	60.5%	54.0%	57.0%
Hypertensive patients with treatment				
Sex				
Male	24.9%	26.0%	21.4%	23.4%
Female	44.0%	41.3%	41.6%	44.2%
Age				
15–24	4.9%	6.2%	4.7%	8.5%
25–34	9.5%	11.7%	11.7%	10.6%
35–44	22.1%	20.2%	17.6%	18.0%
45–54	28.3%	28.0%	23.7%	26.0%
55–64	40.8%	34.3%	33.9%	36.8%
65–74	44.6%	46.3%	41.2%	47.8%
75+	52.6%	49.6%	52.3%	52.6%
Area				
Urban	38.2%	35.6%	32.4%	34.7%
Rural	26.9%	28.6%	26.5%	26.8%
SES				
Very low	24.3%	23.0%	25.7%	24.9%
Low	29.9%	32.6%	26.5%	26.2%
Middle	30.3%	34.1%	34.0%	29.2%
High	44.0%	33.7%	28.7%	39.7%
Very high	45.1%	46.2%	38.7%	43.2%
Controlled hypertension				
Sex				
Male	6.2%	5.8%	4.8%	3.4%
Female	10.8%	10.3%	10.7%	10.9%
Age				
15–24	3.6%	2.0%	3.1%	3.9%
25–34	4.5%	4.7%	4.9%	3.5%
35–44	6.1%	7.4%	4.4%	4.2%
45–54	7.1%	9.5%	6.9%	5.5%
55–64	12.4%	7.9%	9.3%	8.7%
65–74	10.6%	9.6%	7.9%	10.3%
75+	8.9%	8.0%	11.7%	7.9%
Area				
Urban	8.9%	8.2%	8.0%	7.2%
Rural	7.9%	7.5%	6.3%	6.2%
SES				
Very low	7.1%	6.3%	6.6%	4.8%
Low	7.6%	8.4%	6.9%	6.2%
Middle	7.2%	6.0%	8.9%	6.2%
High	11.1%	7.8%	6.1%	7.3%
Very high	9.7%	11.7%	9.6%	9.8%

The weighted proportion by complex sampling.

*SES* socioeconomic status.

When using the ACC/AHA recommended cut-off point, the percentage of individuals aware of their hypertension decreased by 12.8% (see Supplementary 2).

### Hypertensive patients with treatment

**Fig. 3:**
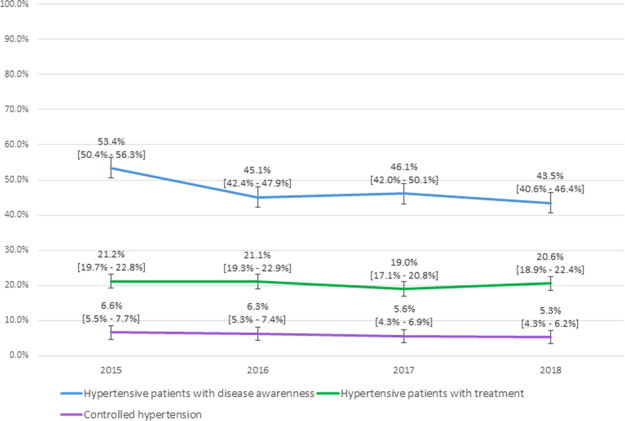
The proportion of Peruvian hypertensive patients with disease awareness, treatment, and controlled hypertension (2015–2018). The weighted proportion by complex sampling.

When analyzing only patients with hypertension, one in five people with hypertension in Peru reported receiving treatment, and no significant trend was found between 2015 and 2018 (score test for trend: *p* value = 0.13, see Fig. 3). In a sub-analysis considering only hypertensive patients with disease awareness, the proportion of those receiving treatment did not change between 2015 and 2018 (36.7% to 42.7%, respectively; score test for trend: *p* value = 0.10). Hypertensive individuals with a high socioeconomic level and older adults had the highest proportion of treatment (see Table 2). The regions located in the Southeastern area of Peru had the lowest proportion of treatment (see Fig. 2c).

### Controlled hypertension

In Peru, only one out of every 20 people with hypertension had their blood pressure controlled, and there was a negative trend over time (score test for trend, *p* value = 0.01). When performing a sub-analysis in only the people receiving treatment, the proportion of people with controlled hypertension varied between 42.1% (2015) and 35.4% (2018; score test for trend, *p* value = 0.03). Men, adolescents, young, and people with a very low economic status with hypertension had the poorest control (see Table 2). Only in three of the 25 regions of Peru, the proportion of patients with controlled hypertension was greater than 10% (see Fig. 2d).

When utilizing the ACC/AHA recommended cut-off point, it halved the proportion of controlled hypertensives (see Supplementary 2).

## Discussion

### Main findings

In Peru, from 2015 to 2018, the age-standardized prevalence of hypertension increased, while the proportion of people with disease awareness and controlled hypertension decreased. During that same period, the proportion of people with treatment for hypertension did not vary over time.

People with low SES and people living in rural areas have the lowest proportion of awareness, treatment, and control of hypertension. Approximately one in five people in Peru have hypertension. This high prevalence coupled with low rates of awareness, treatment, and control of hypertension as well as the deep socioeconomic inequalities throughout the country, constitute a public health problem in Peru.

### Comparison with previous studies

#### Prevalence

The high blood pressure is decreasing across the LMIC [5], and the prevalence of hypertension in several high-income countries [6]. However, the political and health conditions of each country are very heterogeneous, making country-level evaluations necessary. Some countries, such as China [11] and Chile [22], are reporting a trend of an increase in the prevalence of hypertension. In other places, such as Portugal, hypertension is becoming less common and has a downward trend [23]. However, most studies indicate that in other places, hypertension prevalence remains stable over time such as in Italy [24], Mexico [12], Brazil [25], and USA [26].

The differences between national-level estimates (i.e. including only one country) and global trends of several countries may be due to different reasons. First, global trends studies consider a greater amount of time (40 years) [6] compared to the country-level studies (between 5 and 15 years). Second, country-level studies use different methodologies to assess blood pressure. Therefore, it does not allow some studies to be directly compared. When evaluating the age-standardized prevalence of hypertension in Peru in 2018, this estimate was similar to other studies recently published in Peru (19.2% to 19.7%) [27, 28]; nevertheless, the prevalence of hypertension is slightly lower than the average of the LMIC (22%) [4].

The increased hypertension prevalence could be since Peru has experienced a continuous economic growth in recent years and is gradually catching up with the prosperity of other countries in the region. This situation could cause the population to experience an epidemiological transition, as they would be prone to participate in risk behaviors (i.e., smoking, obesity, sedentary) as they would have higher incomes [29]. Our data reinforces this hypothesis since people of lower SES are those who show a greater increase in the age-standardized prevalence of hypertension.

#### Awareness, treatment, and control

When comparing our findings with other similar studies, LMIC present heterogeneous realities. In Latin America, a representative study for Chile (1988–2004) suggests that the percentage of people with disease awareness remains stable over time, but the percentage of people with treatment and control has increased [22]; in Mexico between 2000 and 2012, awareness, treatment, and control are not changing [12]. On the other hand, in Asia, national studies in China (1982–2010) and Korea (2007–2011) suggest that awareness, treatment, and control are increasing [9, 10]. This heterogeneity may be due to the different political conditions and the percentage of resources in the health system of the LMIC.

Decreasing awareness may be explained by the disarticulation of health strategies to detect new cases of hypertension as the Peruvian health system is fragmented [30]. In Peru, all primary care facilities offer free blood pressure screenings but in a passive approach. So, there is a need of increasing the number of activities where health workers search people in the community for blood pressure assessments. Also, educational programs that provide information about the effects of hypertension and the importance of its assessment are needed. There is evidence to show that educational programs in community settings enhance the proportion of hypertensive people mindful of their condition [31]. On the other hand, Peru is experiencing an epidemiological transition that increases the amount of cases of individuals with chronic conditions, which would obligate the health system to detect and treat more users than in the capacity to deal. The Peruvian health system is overburdened by different factors, including limited health personnel and medical equipment [30]. Another reason for the reduction in the proportion of awareness hypertensive is socioeconomic factors, as many poor people cannot afford to invest several hours (for one or several days) to receive medical appointments, attend consultations and follow-ups. That is consistent with our data as people living in rural areas and the poorest have a lower proportion of awareness of their hypertension.

The limited availability of medicines prescribed to hypertensive cases and the out-of-pocket costs associated with the specific treatment [32], could explain the reduction in the rate of controlled hypertension. The poorest would have greater problems accessing treatment because medications may be costly or unavailable within the health system. Economic factors may explain why the Southeastern provinces of Peru have a lower rate of hypertensive with treatment, as they are poorer compared to the coastal regions. Another potential reason for the low proportion of controlled hypertension would be beliefs about hypertension and the lack of knowledge of patients about the health complications presented by the lack of treatment [33]. Some patients discontinue treatment when they feel they have improved (no symptoms) or use irregularly the medicines based on the occurrence of symptoms [33].

The ACC/AHA 2017 clinical practice guidelines recommend lower cut-off points to diagnose hypertension (SBP ≥ 130 mmHg or DBP ≥ 80 mmHg) [21]. The new cut-off would double the age-standardized prevalence of hypertension in Peru as previously showed [34]. Again, this change in a cut-off of hypertension would reduce the percentage of individuals aware, treated, and controlled in Peru, emphasizing the need for proper health strategies to improve these indicators at the population level. The ACC/AHA 2017 clinical practice guidelines recommend the use of indexes to estimate the risk of cardiovascular disease as a fundamental factor in the clinical environment and to guide treatment, particularly in older patients [35]. However, a Peruvian study identified little concordance between different indexes of cardiovascular disease risk (AHA/ACC, Framingham risk score for CVD, Reynolds risk score, systematic coronary risk evaluation, World Health Organization risk charts) [36]. As a result, there is uncertainty in choosing any of them to apply within the Peruvian public health system.

The proportion of awareness, treatment, and control of hypertension vary significantly among LMIC, and data regarding hypertension epidemiology in LMIC are scarce compared to high-income countries. This may be due to factors such as investment in health and the ability to manage health resources, as not all LMIC allocate a sufficient proportion of their resources in health or optimally manage their resources [37]. The World Health Organization proposes that 6% of the gross domestic product be destined for health, but not all LMIC comply with this recommendation, as is the case in Peru [37, 38]. Also, social factors such as poverty, low education of the population, and cultural elements likely also influence hypertension awareness, treatment, and control. Studies support that poverty and inequality in the distribution of resources are some of the main barriers to accessing medical care [39].

Our study presented differences with other studies conducted in Peru on awareness, treatment, and control in hypertensive people. A study conducted throughout Peru identified similar values of people aware of having hypertension (48.2%). However, the values for people with hypertension who are treated (39.3%) and are controlled (20.6%) were higher [40]. Another study conducted in three Peruvian cities (urban and rural) also found higher values for both awareness (69%, 95% CI 64.4–73.5%), self-reported treatment (63.2%, 95% CI 58.6–67.8%) and control (45.9%, 95% CI 40.9–50.8%) [27]. On the other hand, another study conducted in three Peruvian cities with migrants and non-migrants identified values similar to those reported in our study for awareness (48.3%), treatment (16.6%), and control of hypertension (4.9%) [41]. These differences may be due to different methodologies or heterogeneous populations. Nonetheless, our results provide a better picture and stronger results to inform policies and interventions because we have benefitted from a national sample and data over 4 years.

### Relevant in public health

Health policies and interventions must be established to reduce the prevalence and increase the proportion of awareness, treatment, and control of hypertension since it is a public health problem for Peru.

First, strengthen national strategies for screening and diagnosis of hypertension. It is necessary to increase the proportion of hypertensive people with disease awareness because awareness is a basic condition for receiving treatment and achieving blood pressure control. It is suggested that these strategies should have an emotional support component for newly diagnosed people, because there is evidence that people who receive a diagnosis of hypertension are twice as likely to have depressive symptoms, compared to people without hypertension or hypertension without awareness of the disease [42]. Second, to develop decentralized strategies for the care and diagnosis of hypertension cases and to actively involve the community. Evidence suggests active involvement of local communities improves the outcomes of hypertensive patient management and their control of hypertension [43]. Third, establish a surveillance system to identify people with hypertension and send them regular reminders (e.g. text messages) to improve their monitoring and treatment. These interventions are more effective for treatment adherence than attending primary care within the health system [44]. Fourth, establish periodic surveillance of the prevalence, awareness, treatment, and control of hypertension to evaluate the effect of implemented strategies.

### Strengths and limitations

The present study was carried out in a nationally representative sample of the Peruvian population. However, this study has limitations. First, the international guidelines suggest three blood pressure measurements and discard the first due to the white coat effect [16]. Because we have only two blood pressure measurements, results may be altered. Therefore, a set of criteria was used to assess the plausibility of blood pressure measurements, based on evaluating the difference between the two measurements and identifying measurements with less than ≤5 mmHg between them. Second, the criteria used in our study to assess the plausibility of the measurements may alter the trend results (our study used a mixed three-step criterion). To evaluate this possibility, three additional analyses were performed where trends were evaluated using a criterion where the difference between the two SBP measurements and the two BPD measurements was ≤5 mmHg, ≤10 mmHg, and ≤15 mmHg. These three analyses were consistent and did not change our main trend results. Therefore, we do not consider the criterion used to assess the plausibility of the data to be a factor that could be modifying the results. Third, the evaluation time may be insufficient to identify a clear trend, and for instance, further longer surveillance is needed. Finally, other relevant variables that could support a further understanding of the results were not applicable. So, information on treatment adherence, the treatment received, secondary forms of hypertension (i.e., primary aldosteronism, Cushing Syndrome, Pheochromocytoma), and other sociodemographic variables (i.e., ethnic background) was not included in the analyses as they were not available.

## Conclusions

In Peru, between 2015 and 2018, a sustained increase over time in age-standardized prevalence, a reduction in the proportion of people aware and with controlled hypertension was identified. The proportion of individuals with hypertension under treatment remained stable during this period (4 years).

## Summary

### What is known about the topic

- The prevalence of high blood pressure has been declining in low- and middle-income countries (LMIC), particularly in Latin America and the Caribbean.
- There are limited studies evaluating trends for awareness, treatment, and control of hypertension in LMICs.
- Different national studies carried out in LMIC identify heterogeneity in the trends of knowledge, treatment, and control of hypertension.

### What this study adds

- An increase in hypertension prevalence and a reduction in awareness and control of hypertension are identified in Peru.
- People with low socioeconomic status and people living in rural areas have the lowest proportion of awareness, treatment, and control of hypertension.

## Supplementary information

Supplement 1

Supplement 2
