# Supplementary material for: Short-term trends in the prevalence, awareness, treatment, and control of arterial hypertension in Peru
Source: J Hum Hypertens. 2020 Jun 9;35(5):462–71. doi: 10.1038/s41371-020-0361-1 (PMC8134053; doi:10.1038/s41371-020-0361-1)
Supplement: Supplementary file 1 — Supplement 1 [file 41371_2020_361_MOESM1_ESM.docx]

**Supplement 1. Descriptive characteristics of the participants not included in the study.**

|  |  | 2015 |  |  | 2016 |  |  | 2017 |  |  | 2018 |  |  |
| --- | --- | --- | --- | --- | --- | --- | --- | --- | --- | --- | --- | --- | --- |
|  |  | No Include | | Include | No Include | | Include | No Include | | Include | No Include | | Include |
|  |  | n | % | % * | n | % | % * | n | % | % * | n | % | % * |
| Sex | Male | 2,572 | 43.2% | 48.9% | 2,446 | 43.0% | 49.1% | 2,613 | 43.0% | 48.4% | 2,692 | 42.7% | 48.3% |
|  | Female | 3,386 | 38.0% | 51.1% | 3,247 | 37.7% | 50.9% | 3,464 | 37.7% | 51.6% | 3,617 | 37.2% | 51.7% |
|  | Missing | 1,101 | - | - | 1,166 | - | - | 880 | - | - | 912 | - | - |
| Age | 15-24 | 1,363 | 22.9% | 27.6% | 1,241 | 21.8% | 26.3% | 1,314 | 21.6% | 24.6% | 1,332 | 21.1% | 24.4% |
|  | 25-34 | 1,707 | 28.7% | 20.7% | 1,568 | 27.5% | 20.5% | 1,768 | 29.1% | 21.9% | 1,714 | 27.2% | 20.9% |
|  | 35-44 | 1,259 | 21.1% | 18.9% | 1,205 | 21.2% | 18.3% | 1,214 | 20.0% | 18.6% | 1,367 | 21.7% | 18.4% |
|  | 45-54 | 684 | 11.5% | 14.0% | 663 | 11.7% | 15.1% | 706 | 11.6% | 14.6% | 774 | 12.3% | 15.6% |
|  | 55-64 | 461 | 7.7% | 8.8% | 470 | 8.3% | 8.9% | 508 | 8.4% | 10.0% | 529 | 8.4% | 9.8% |
|  | 65-74 | 287 | 4.8% | 5.8% | 325 | 5.7% | 6.6% | 324 | 5.3% | 5.8% | 364 | 5.8% | 6.7% |
|  | 75+ | 197 | 3.3% | 4.2% | 221 | 3.9% | 4.4% | 243 | 4.0% | 4.6% | 229 | 3.6% | 4.3% |
|  | Missing | 1,101 | - | - | 1,166 | - | - | 880 | - | - | 912 | - | - |
| Area | Urban | 4,731 | 67.0% | 65.5% | 4,628 | 67.0% | 64.80% | 4,631 | 67.0% | 79.50% | 4,814 | 67.0% | 80.40% |
|  | Rural | 2,328 | 33.0% | 34.6% | 2,231 | 33.0% | 35.20% | 2326 | 33.0% | 20.50% | 2407 | 33.0% | 19.60% |
| SES | Very low | 1,963 | 27.8% | 26.2% | 1,812 | 26.4% | 26.30% | 2,087 | 30.0% | 18.30% | 2,219 | 30.8% | 18.70% |
|  | Low | 1,694 | 24.0% | 21.3% | 1,668 | 24.3% | 22.20% | 1,739 | 25.0% | 20.70% | 1,697 | 23.5% | 20.80% |
|  | Middle | 1,347 | 19.1% | 18.3% | 1,350 | 19.7% | 18.50% | 1,282 | 18.4% | 21.00% | 1,346 | 18.7% | 20.70% |
|  | High | 1,134 | 16.1% | 17.9% | 1,126 | 16.4% | 17.40% | 1,076 | 15.5% | 20.30% | 1,077 | 14.9% | 20.30% |
|  | Very High | 921 | 13.0% | 16.3% | 903 | 13.2% | 15.70% | 773 | 11.1% | 19.80% | 882 | 12.2% | 19.50% |

Note: SES = Socioeconomic status; * The weighted proportion by complex sampling.
