# Supplementary material for: Short-term trends in the prevalence, awareness, treatment, and control of arterial hypertension in Peru
Source: J Hum Hypertens. 2020 Jun 9;35(5):462–71. doi: 10.1038/s41371-020-0361-1 (PMC8134053; doi:10.1038/s41371-020-0361-1)
Supplement: Supplementary file 2 — Supplement 2 [file 41371_2020_361_MOESM2_ESM.docx]

**Supplement 2. Comparison and difference between two cut-off points for hypertension.**

|  |  | 2015 | 2016 | 2017 | 2018 | *p* |
| --- | --- | --- | --- | --- | --- | --- |
| Cut-off | Age-standardized prevalence * | 18.7% | 18.5% | 19.4% | 20.6% | < 0.001 |
| SBP ≥ 140 mmHg or DBP ≥ 90 mmHg | Hypertensive patients with disease awareness ** | 53.40% | 45.10% | 46.1% | 43.5% | < 0.001 |
|  | Hypertensive patients with treatment ** | 21.2% | 21.1% | 19.0% | 20.6% | 0.130 |
|  | Controlled hypertension ** | 6.6% | 6.3% | 5.6% | 5.3% | 0.010 |
| Cut-off (AHA/ACC 2017) | Age-standardized prevalence * | 32.3% | 32.1% | 33.0% | 36.6% | < 0.001 |
| SBP ≥ 130 mmHg or DBP ≥80 mmHg | Hypertensive patients with disease awareness ** | 34.8% | 31.9% | 31.1% | 30.7% | < 0.001 |
|  | Hypertensive patients with treatment ** | 20.2% | 19.4% | 18.5% | 19.0% | 0.012 |
|  | Controlled hypertension ** | 3.6% | 3.3% | 3.3% | 2.7% | < 0.001 |
| Difference | Age-standardized prevalence * | +13.6% | +13.6% | +13.6% | +16.0% | - |
|  | Hypertensive patients with disease awareness ** | -18.6% | -13.2% | -15.1% | -12.8% | - |
|  | Hypertensive patients with treatment ** | -1.0% | -1.7% | -0.5% | -1.6% | - |
|  | Controlled hypertension ** | -3.1% | -3.0% | -2.3% | -2.6% | - |

*Note:* * denominator is the population of Peru for each year ** denominator is the number of hypertensive for each year. *p* = p value of score test for trend. ACC/AHA = American College of Cardiology/American Heart Association.
